# Culprit-Lesion Drug-Coated-Balloon Percutaneous Coronary Intervention in Patients Presenting with ST-Elevation Myocardial Infarction (STEMI)

**DOI:** 10.3390/jcm14030869

**Published:** 2025-01-28

**Authors:** Jorge Sanz-Sánchez, Andrea Teira Calderón, David Neves, Carlos Cortés Villar, Antonela Lukic, Eva Rumiz González, Guillermo Sánchez-Elvira, Lino Patricio, José Luis Díez-Gil, Héctor M. García-García, Luis Martínez Dolz, J. Alberto San Román, Ignacio Amat Santos

**Affiliations:** 1 Hospital Universitari i Politecnic La Fe, 46026 Valencia, Spain; sjorge4@gmx.com (J.S.-S.); diez_jlu@gva.es (J.L.D.-G.); luismartinezdolz@gmail.com (L.M.D.); 2 Centro de Investigación Biomedica en Red (CIBERCV), 28029 Madrid, Spain; asanroman@secardiologia.es; 3 Hospital Espírito Santo, 7000-811 Évora, Portugallinopatricio@gmail.com (L.P.); 4 Hospital Clínico Universitario de Valladolid, 47003 Valladolid, Spain; carlos.cortes.villar@gmail.com; 5 Hospital Clínico Universitario Lozano Blesa, 50009 Zaragoza, Spain; antonelalukic@yahoo.es; 6 Hospital General Universitario de Valencia, 46014 Valencia, Spain; evarumizgonzalez@gmail.com; 7 Hospital Universitario de Navarra, 31006 Pamplona, Spain; guillermo.sanchez.elvira@navarra.es; 8 MedStar Washington Hospital Center, Washington, DC 20010, USA; hect2701@gmail.com

**Keywords:** drug-coated balloon, percutaneous coronary intervention, ST-elevation myocardial infarction

## Abstract

**Background/Objectives**: Drug-eluting stents (DESs) remain the standard of treatment for patients with ST-elevation myocardial infarction (STEMI). However, complications such as stent thrombosis and in-stent restenosis still pose significant risks. Drug-coated balloons (DCBs) have emerged as a promising alternative, but data for this clinical scenario are still scarce. The objective was to evaluate the safety and efficacy of DCB culprit-lesion primary percutaneous coronary intervention (pPCI) in patients presenting with STEMI and to evaluate its impact on the microcirculatory territory. **Methods**: An observational retrospective study was conducted across six European centers. **Results**: In total, 118 patients were included. Of these, 82.2% were male, with a median age of 67 years (IQR 36–92); 28% patients presented with stent thrombosis and most of them (94%) underwent paclitaxel-DCB-pPCI. The median follow-up was 23.2 months (IQR 6.7–77.3). Target lesion failure (TLF) rates were low (3.4%), with no differences between patients presenting with native coronary vessel and stent thrombosis (4.7% vs. 0%; *p* = 0.205). Overall mortality rates at follow-up were 7%, with only 1.8% attributed to cardiac causes. A target lesion revascularization (TLR) rate of 1.8% was observed, with no target vessel myocardial infarction reported. A subgroup of patients (42; 35.6%) underwent an adenosine-free angiographic microvascular resistance (AMR) analysis. The median AMR was 4.7 (3.9–5.5) and was greater in the stent thrombosis group than in the native coronary group (5.1 vs. 4.6; *p* = 0.038) with no clinical differences between patients based on the AMR. **Conclusions**: DCB-pPCI has emerged as an alternative potential treatment for patients presenting with STEMI, with few long-term adverse cardiac events. Despite the encouraging outcomes, these findings underscore the need for a large randomized clinical trial powered by a relevant clinical outcome in order to elucidate the role of DCB-PCI in patients presenting with STEMI.

## 1. Introduction

In patients with ST-segment elevation myocardial infarction (STEMI), the recommended treatment is primary percutaneous coronary intervention (pPCI) [1,2]. Traditionally, pPCI has involved the implantation of drug-eluting stents (DESs) [3]. However, this approach is associated with adverse cardiac events related to stent placement such as in-stent restenosis and stent thrombosis [4,5]. In this setting, drug-coated balloons (DCBs) have been developed as a potential alternative [6]. DCBs are balloon catheters coated with an antiproliferative agent that is released into the vessel wall upon balloon inflation. The absence of a permanent metallic scaffold may help to preserve vessel vasomotion and potentially reduce the risk of long-term adverse events [7,8,9,10]. On the other hand, DCB-PCI might lead to acute recoil, coronary dissection and vessel closure. In addition, the multiple vessel predilations required during DCB-PCI might lead to microvascular disfunction, reduced TIMI flow and adverse clinical outcomes. DCBs might also pose certain risks in the setting of STEMI patients as acute recoil with closure of the culprit lesion due to the lack of vessel scaffolding. However, these risks remain theoretical, with no evidence in the published literature. In addition, data regarding the role of DCB-PCI in patients with STEMI are very limited [11,12,13] and focus on single-center reports and native coronary vessels. Therefore, the aim of this study was to evaluate the safety and efficacy of DCB-pPCI in all-comer patients presenting with STEMI in the setting of a real-world European multicenter registry, with a focus on the impact of DCB-PCI on the microcirculation territory.

## 2. Methods

### 2.1. Study Design

The present study was a retrospective multicenter study that included STEMI patients who underwent culprit-lesion pPCI with DCB across six European centers from March 2013 to February 2024.

The inclusion criteria were broad and included (1) patients presenting with STEMI and an indication to undergo pPCI and (2) patients undergoing culprit-lesion DCB-pPCI. The exclusion criteria were (1) patients in cardiogenic shock and (2) patients undergoing culprit-lesion DES PCI.

The central ethics committee and local ethics committee at each participating center approved the study protocol.

### 2.2. Outcomes

The primary endpoint was target lesion failure (TLF), defined as a composite of cardiovascular death, clinically driven target lesion revascularization (TLR) and target vessel myocardial infarction (MI). Secondary endpoints included cardiac death, target vessel MI, non-target-vessel MI, TLR, target vessel revascularization, acute kidney injury and BARC (Bleeding Academic Research Consortium) bleeding. All the aforementioned endpoints were defined according to Academy Research Consortium criteria [14] with the exception of MI, which was defined according to the fourth universal definition [15]. All outcomes were compared between patients presenting with native vessel STEMI and those presenting with stent thrombosis STEMI.

In addition, we performed a subgroup analysis of patients for whom coronary angiographies were available. In those patients, we evaluated the microcirculation territory using an adenosine-free angiographic microvascular resistance analysis following DCB-pPCI using Angioplus^®^ software, version 2.1.1.0 (Shanghai Pulse Medical Technology, INC, Shanghai, China). This computational method uses artificial intelligence to estimate the AMR based on the analysis of a single angiographic projection, with excellent reproducibility [16]. Briefly, one angiographic projection with minimal overlap was selected. The entire treated vessel was analyzed. The software automatically detected the vessel contours and estimated the AMR. We used a recently validated equation that correlates the AMR and IMR (index of microvascular resistance) as follows: AMR = 0.90 + 0.07 × IMR [16,17]. Using this formula, we set an AMR cutoff value of 3.7 for our analysis, corresponding with an IMR of 40 that has previously been associated with adverse clinical outcomes and more extensive myocardial injury in patients presenting with STEMI [18].

### 2.3. Statistical Analysis

The continuous variables were expressed as the mean with a standard deviation (SD) or as a median with an interquartile range (IQR) as appropriate. Categorical variables were expressed as frequencies and percentages. We divided our cohort into two groups based on their clinical presentation (stent thrombosis or native coronary vessel). All comparisons were made using two sample proportion tests. Clinical follow-up was defined as the date of death or the latest available follow-up. Statistical significance was set at a two-tailed *p* < 0.05. All analyses were performed using Stata software, version 16.1 (Station College, TX, USA).

## 3. Results

### 3.1. Study Population and Clinical Characteristics

From March 2013 to February 2024, a total of 118 patients were enrolled across six European centers.

The baseline characteristics of included patients are shown in Table 1. The median age was 67 years (IQR 36–92) and 17.8% were women. There were significantly more female patients presenting with native vessel STEMI than those presenting with stent thrombosis (23.5% vs. 3%; *p* = 0.009). Overall, 40.5% of the patients had a prior history of diabetes mellitus, 52.2% had hypertension, 67% had dyslipidemia and 33.9% were active smokers. In addition, 30.4% of the patients had a history of prior MI. The median left ventricular ejection fraction was 52.8% (IQR 30–74).

### 3.2. Procedural Characteristics

The procedural characteristics are shown in Table 2. A total of 109 (94%) PCIs were performed via trans-radial access. Regarding the angiographic findings, 28% of the cases presented with stent thrombosis (median time from index procedure to stent thrombosis: 1334 days; IQR 232–2269) and 54.7% had multivessel disease. The left anterior descending artery was the most commonly treated vessel (25.4%). The mean reference vessel diameter was 2.6 ± 0.6 mm, with significant differences between patients presenting with native vessel STEMI and those with stent thrombosis (2.5 mm vs. 3.0 mm; *p* = 0.001). The mean lesion length was 17.6 ± 11.1 mm, with no differences between groups (Table 2).

A total of 39 (33.3%) lesions were bifurcations, with a significantly higher proportion in the native vessel group (48.5% vs. 15.2%; *p* = 0.009).

Lesion preparation was performed in 93.2% of the cases prior to DCB-PCI, mainly using semi-compliant balloons (76; 66.1%), followed by cutting balloons (17; 14.8%) and non-compliant balloons (15; 13%). Thromboaspiration was performed in 17% of the cases, with no differences between groups (*p* = 0.824). Intravascular imaging was used significantly more frequently in patients presenting with stent thrombosis than in patients presenting with native vessel STEMI (33.3% vs. 5.9%; *p* = 0.001). Regarding the type of DCB used, 95.6% (111) of the procedures were performed with paclitaxel-DCB and 3.4% (4) with sirolimus-DCB (Table 2).

Coronary dissections after DCB-PCI were reported in eleven cases (9.3%). Five were type A dissections, four were type B and two were type C. Bailout stenting was performed in four cases (3.4%). There was one case of vessel perforation (0.9%), one case of lateral branch occlusion (0.9%) and five cases of slow flow (4.3%). There were numerical, but no statistically significant, differences in the procedural complications (dissection type C, perforation, lateral branch occlusion or slow flow) between patients presenting with native vessel STEMI and those presenting with stent thrombosis STEMI (10.6% vs. 0%; *p* = 0.068).

### 3.3. Clinical Outcomes

**Figure 1:**
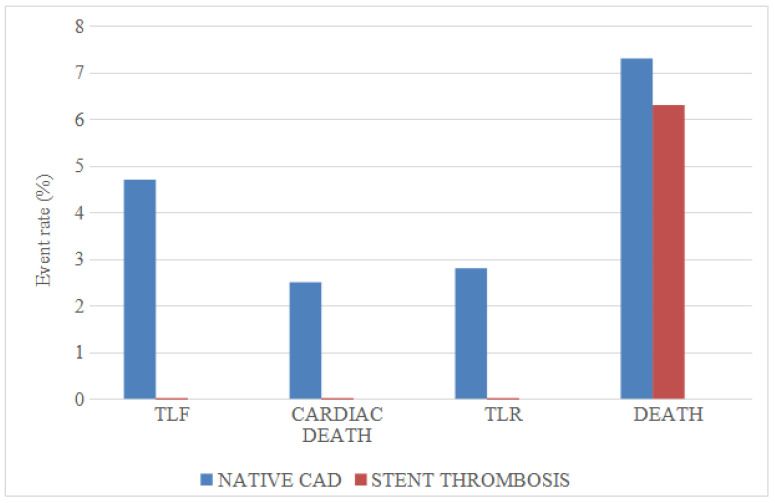
Follow up complications comparing native vessel versus stent thrombosis STEMI. Target lesion failure was defined as a composite of cardiac death, clinically driven TLR and target vessel MI. CAD: coronary artery disease; TLF: target lesion failure; TLR: target lesion revascularization.

The median follow-up was 23.2 months (IQR 6.7–77.3). TLF occurred in four cases (3.5%), with no significant differences between patients presenting with native vessel and stent thrombosis STEMI (4.7 vs. 0%; *p* = 0.205) (Figure 1). The all-cause mortality rate was 7%, with two deaths due to cardiac causes (1.8%). Two cases of clinically driven TLR (1.8%) and four cases of stroke (3.5%) were observed. No target vessel MI was reported. Regarding bleeding events, nine cases of BARC bleeding were reported, two of type 1, four of type 2 and three of type 3. No significant differences for any clinical outcome were observed among patients presenting with native vessel STEMI and those with stent thrombosis. The detailed list of clinical outcomes is presented in Table 3.

### 3.4. Microcirculation Territory

The impact of culprit-lesion DCB-PCI on the microcirculation territory was analyzed in 42 patients (35.6%) for whom coronary angiographies were available. The median AMR was 4.7 (IQR 3.9–5.5) and was greater in patients presenting with stent thrombosis compared with those presenting with native vessel STEMI (5.1 vs. 4.6; *p* = 0.038). No differences were found between patients with either an AMR above or below 3.7. Detailed clinical outcomes based on AMR are shown in Table 4.

## 4. Discussion

The aim of this real-world multicenter registry of patients presenting with STEMI was to assess the safety and effectiveness of DCB-pPCI and evaluate its impact on the microcirculation territory. The main findings of this study can be summarized as follows:

1. Culprit-lesion DCB-PCI in patients presenting with STEMI was associated with low major adverse clinical outcomes.
2. No significant differences for any outcome were found among patients presenting with native vessel STEMI and those presenting with stent thrombosis.
3. Patients undergoing culprit-lesion DCB-PCI for stent thrombosis were associated with higher rates of AMR without any clinical impact compared with patients presenting with native vessel STEMI.

DES-PCI is the standard of treatment for patients presenting with STEMI. However, complications such as stent thrombosis and in-stent restenosis still occur. Despite their relatively low incidence, with rates estimated at 0.7–1% [19,20] for stent thrombosis and 5–10% [21] for in-stent restenosis, these complications can be fatal. In addition, long-term adverse cardiac events in patients presenting with STEMI have been reported to be as high as 32.4% at 10-year follow-up [22].

Evidence regarding the role of DCB-PCI in patients presenting with STEMI is scarce. The Revelation randomized trial, which included 120 STEMI patients, showed that culprit-lesion DCB-PCI was noninferior to DES in terms of the fractional flow reserve at a 9-month follow-up [23]. Merinopoulos et al., in a retrospective propensity score-matching analysis involving 452 patients undergoing culprit-lesion paclitaxel-DCB-PCI, demonstrated that DCB-PCI in patients presenting with STEMI was safe in terms of all-cause mortality and all net adverse cardiac events, including unplanned target lesion revascularization [13]. Of note, both studies were single-center reports and only included patients with native vessel STEMI. It is noteworthy that in our multicenter study from six European centers, up to 28% of patients presented with stent thrombosis, which has been associated with worse clinical outcomes compared with patients presenting with native vessel coronary artery disease [24] and has traditionally been excluded from randomized clinical trials involving patients with STEMI [25,26,27]. Notwithstanding, our findings were in line with the aforementioned evidence, showing very low rates of TLF (3.5%), all-cause mortality (7%) and TLR (2%). Additionally, no significant differences between patients presenting with native vessel and stent thrombosis STEMI for any of the outcomes were observed. Patients presenting with stent thrombosis were characterized by a significantly lower proportion of females (3% vs. 23.5%; *p* = 0.009), a higher incidence of active smokers (54.6% vs. 25.6%; *p* = 0.001) and a higher incidence of prior MI (81.8% vs. 9.8%; *p* = 0.001). Regarding angiographic characteristics, patients presenting with native vessel STEMI included a higher proportion of patients undergoing DCB-PCI for bifurcations (48.5% vs. 15.2%; *p* = 0.009), likely reflecting an operator bias toward a DCB-only approach in such settings. Conversely, patients presenting with stent thrombosis more frequently underwent intravascular-imaging-guided PCI (33.3% vs. 5.9%; *p* = 0.001). In addition, the mean reference vessel diameter in our investigation was 2.6 mm and was significantly larger in the stent thrombosis group compared with the native vessel group (3.0 mm vs. 2.6 mm; *p* = 0.001), while in the Revelation trial and in the Merionopoulos et al. study, the mean reference vessel diameter was 3.28 mm and 3.5 mm, respectively. This discrepancy might also contribute to the differences observed in the rates of clinical outcomes among different studies [24].

Regarding the microcirculation territory, IMR remains the most used tool to assess the coronary microcirculation status [28]. Previous published studies have shown an association between increased IMR values and adverse clinical outcomes in both obstructive and non-obstructive coronary artery disease [29,30,31,32]. El Farissi et al., in an individual patient data-pooled analysis including 1265 patients, reported that patients with an IMR > 40 had significantly higher rates of cardiac death (4.9% vs. 2.2%) [33] compared with those patients with an IMR < 40. Scarsini et al., in a retrospective analysis involving 568 STEMI patients, reported a good correlation between angiography-derived and wire-based IMRs. An IMR cutoff value ≥ 40 was associated with a higher incidence of adverse cardiovascular events [34]. Furthermore, microvascular obstruction has been reported to be an independent predictor of adverse cardiac events [35]. This is particularly relevant in patients presenting with STEMI, as predilation and consequent distal embolization can lead to microvascular obstruction; therefore, direct stenting has been proposed as an alternative strategy this setting [36,37,38]. During DCB-pPCI, predilation is crucial in order to achieve proper lesion preparation to facilitate subsequent drug absorption. However, this might lead to the mechanical fragmentation of plaques or thrombotic material, distal embolization downstream of the infarct-related artery, microvascular obstruction and adverse clinical outcomes. However, this remains theoretical as no previous investigation has assessed the impact of DCB-PCI in patients presenting with STEMI. Therefore, we decided to evaluate the impact of DCB-PCI on the microcirculation territory using an angiography-derived index provided by Angioplus^®^ software, version 2.1.1.0 (Shanghai Pulse Medical Technology, INC). Fan et al. recently evaluated this software using 257 vessels and demonstrated a good correlation (r = 0.83; *p* < 0.001) and diagnostic performance (AUC 0.94; 95% CI: 0.91 to 0.97) compared with a wire-based IMR [17]. An AMR ≥ 3.7 corresponds with an IMR ≥ 40 [28]. Therefore, we selected this threshold as it has previously been associated with worse clinical outcomes in STEMI patients [18,34]. In our investigation, the median AMR immediately after DCB-pPCI was 4.7 (3.9–5.5). Most of the patients (34; 81%) had an AMR value ≥ 3.7 immediately post pPCI. This finding was consistent with prior data, where the mean IMR in STEMI patients was 39.5 [34], corresponding with an AMR of 3.7 [17]. The post-DCB-PCI AMR was significantly higher in patients presenting with stent thrombosis compared with patients with native vessel STEMI (5.1 vs. 4.6; *p* = 0.038), which might be explained by the more aggressive predilation performed on this population. Despite a higher microvascular disfunction in patients presenting with stent thrombosis, no differences for any clinical outcomes were found between groups. Notwithstanding, these results should be considered to be hypothesis-generating given the limited sample size and reduced number of adverse events at follow-up.

In conclusion, despite promising results with culprit-lesion DCB-PCI in patients presenting with STEMI, a large, powered, randomized clinical trial to evaluate the role of DCB revascularization in this population is required. Such a study (NCT06353594) is currently ongoing and will provide solid evidence for this scenario.

## 5. Study Limitations

The present study should be interpretated in light of some limitations. First, this was was a retrospective observational study, which has limitations and biases inherent in its nature. In addition, the period inclusion was long, with few patients included per center. Second, the event rate in the present study was low, limiting the possibility of achieving conclusive comparations between patients presenting with native vessel STEMI and those presenting with stent thrombosis STEMI. Third, the event adjudication was performed by the study investigators. Fourth, the study included patients treated using different DCBs. Therefore, heterogeneity in the management and underlying conditions might have introduced variability into the treatment effects. Fifth, as AMR relies on angiography, its accuracy depends on the quality of images. To minimize this limitation, we only considered optimal angiographic images for the analysis.

## 6. Conclusions

The results herein presented highlight the potential role of DCB-pPCI in patients presenting with STEMI, with low rates of adverse clinical events. However, this study underscores the imperative need for a large, clinically powered randomized trial to comprehensively address the role of DCB-PCI in STEMI.

## Figures and Tables

**Table 1:** Baseline characteristics and clinical presentation based on native vessel or stent thrombosis disease.

Baseline Characteristics				
	Overall (*n* = 118)	Native Vessel (*n* = 85)	Stent Thrombosis (*n* = 33)	*p*-Value
Female, *n* (%)	21 (17.8)	20 (23.5)	1 (3)	0.009
Age, Median (IQR)	67.5 (36–92)	67.5 (36–92)	67.4 (36–88)	0.981
Diabetes Mellitus, *n* (%)	32 (40.51)	18 (31.6)	14 (63.6)	0.006
Hypertension, *n* (%)	60 (52.2)	41 (50)	19 (576)	0.462
Dyslipidemia, *n* (%)	77 (67)	51 (62.2)	26 (78.8)	0.087
Current Smoker, *n* (%)	39 (33.9)	21 (25.6)	18 (54.6)	0.005
Atrial Fibrillation	12 (10.4)	9 (11)	3 (9.1)	0.765
COPD, *n* (%)	6 (5.2)	3 (3.7)	3 (9.1)	0.236
Prior MI, *n* (%)	35 (30.4)	8 (9,8)	27 (81.8)	0.001
Prior PCI, *n* (%)	39 (33.9)	10 (12.2)	33 (100)	0.001
Prior Stroke, *n* (%)	7 (5.8)	4 (4.9)	3 (9,4)	0.369
Prior Peripheral Artery Disease, *n* (%)	4 (3.5)	1 (1.2)	3 (9.1)	0.037
Prior Bleeding, *n* (%)	3 (2.5)	1 (1.2)	2 (6.1)	0.141
Prior Liver Disease, *n* (%)	5 (4.3)	2 (2.4)	3 (9.1)	0.114
LVEF, Median (IQR)	52.8 (30–74)	54 (33.2–74)	49.5 (30–70)	0.031
GFR, Mean ± SD	75.1 ± 28.6	76.8 (30–70)	70.6 (41–90)	0.170

Values are *n* (%), mean SD or median with interquartile range. COPD: chronic obstructive pulmonary disease; GFR: glomerular filtration rate; IQR: interquartile range; LVEF: left ventricular ejection fraction; MI: myocardial infarction; PCI: percutaneous coronary intervention; SD: standard deviation.

**Table 2:** Procedural characteristics and treatment at discharge based on native vessel or stent thrombosis disease.

Procedure Characteristics				
	Overall (*n* = 118)	Native Vessel (*n* = 85)	Stent Thrombosis (*n* = 33)	*p*-Value
Radial Access, *n* (%)	109 (94)	81 (96.4)	28 (87.5)	0.071
Multivessel Disease, *n* (%)	64 (54.7)	44 (52.4)	20 (60.6)	0.421
Treated Vessel				
LM, *n* (%)	2 (1.7)	1 (1.2)	1 (3)	0.003
LAD, *n* (%)	30 (25.4)	14 (16.5)	16 (48.5)	
Diagonal, *n* (%)	24 (20.3)	22 (25.9)	2 (6.1)	
Circumflex, *n* (%)	10 (8.5)	6 (7.1)	4 (12.1)	
OM, *n* (%)	14 (11.9)	11 (13)	3 (9.1)	
RI, *n* (%)	7 (5.9)	7 (8.2)	0 (0)	
RCA, *n* (%)	20 (17)	13 (15.3)	7 (21.2)	
PDA, *n* (%)	6 (5.1)	6 (7.1)	0 (0)	
RPB, *n* (%)	5 (4.2)	5 (5.9)	0 (0)	
Bifurcation, *n* (%)	39 (33.3)	34 (48.5)	5 (15.2)	0.009
Severe Calcification, *n* (%)	5 (4.2)	3 (3.5)	2 (6.1)	0.540
Thromboaspiration, *n* (%)	20 (17)	14 (16.5)	6 (18.2)	0.824
Intracoronary Imaging	16 (13.6)	5 (5.9)	11 (33.3)	0.001
IVUS, *n* (%)	9 (7.6)	3 (3.5)	6 (18.2)	
OCT, *n* (%)	7 (5.9)	2 (2.4)	5 (15.2)	
Reference Vessel Diameter (mm), Mean ± SD	2.6 ± 0.6	2.5 ± 0.5	3 ± 0.5	0.001
Lesion Length (mm), Mean ± SD	17.6 ± 11.1	16.6 ± 11.5	20 ± 9.9	0.143
Predilation				0.001
Semi-Compliant Balloon, *n (*%)	75 (66.1)	62 (73.8)	13 (42)	
Non-Compliant Balloon, *n (*%)	15 (13)	7 (8.3)	8 (25.8)	
Cutting Balloon, *n (*%)	17 (14.8)	9 (10.7)	8 (25.8)	
Scoring Balloon, *n (*%)	2 (1.7)	0 (0)	2 (6.5)	
DCB Type				0.369
Pantera Lux	33 (28.2)	27 (32.5)	6 (18.8)	
Prevail	14 (12)	9 (10.8)	5 (15.6)	
SeQuent Please	34 (29.1)	26 (31.3)	8 (25)	
Agent	29 (24.8)	18 (21.7)	11 (34.4)	
Magictouch	4 (3.5)	3 (3.6)	1 (3.1)	
Everflow	1 (0.9)	0 (0)	1 (3.1)	
DCB Diameter (mm), Mean ± SD	2.6 ± 0.6	2.5 ± 0.5	2.9 ± 0.6	0.001
Number of DCBs, Mean (IQR)	1.1 (1–3)	1 (1–3)	1.1 (1–3)	0.256
Dissection After DCB				0.395
Type A, *n (*%)	5 (4.2)	5 (6)	0 (0)	
Type B, *n (*%)	4 (3.4)	3 (3.6)	1 (3)	
Type C, *n (*%)	2 (1.7)	2 (2.4)	0 (0)	
Complications After DCB	9 (7.6)	9 (10.6)	0 (0)	0.068
Dissection Type C, *n (*%)	2 (1.7)	2 (2.4)	0 (0)	
Perforation, *n* (%)	1 (0.9)	1 (1.2)	0 (0)	
Lateral Branch Occlusion, *n* (%)	1 (0.9)	1 (1.2)	0 (0)	
Slow Flow, *n* (%)	5 (4.3)	5 (6)	0 (0)	
TIMI Flow After DCB, Mean ± SD	2.9 ± 0.4	2.8 ± 0.5	3 ± 0.2	0.152
DES Bailout, *n* (%)	4 (3.4)	4 (4.7)	0 (0)	0.205
Final AMR, Median (IQR)	4.7 (3.9–5.5)	4.6 (3.6–5.2)	5.1 (4.7–7.5)	0.038
AMR ≥ 3.7, *n* (%)	34 (81)	24 (75)	10 (100)	0.079
**Treatment at Discharge**				
Aspirin, *n* (%)	111 (97.4)	79 (97.5)	32 (97)	0.865
Clopidogrel, *n* (%)	42 (36.8)	31 (38.3)	11 (33.3)	0.620
Prasugrel, *n* (%)	17 (14.9)	13 (16.1)	4 (12.1)	0.593
Ticagrelor, *n* (%)	54 (47.4)	36 (44.4)	18 (55.6)	0.327
Anti-Vitamin K, *n* (%)	2 (1.8)	1 (1.3)	1 (3)	0.527
DOAC, *n* (%)	15 (13.4)	11 (13.9)	4 (12.1)	0.798

Values are *n* (%), mean SD or median with interquartile range. AMR: angiographic microvascular resistance; DCB: drug-coated balloon; DES: drug-eluting stent; DOAC: direct-acting oral anticoagulant; LAD: left anterior descending; LM: left main; OM: obtuse marginal; PDA: posterodescendent artery; RCA: right coronary angiography; RI: ramus intermedius; RPB: right posterolateral branch.

**Table 3:** Outcomes during follow-up based on native vessel or stent thrombosis disease.

	Overall (*n* = 118)	Native Vessel (*n* = 85)	Stent Thrombosis (*n* = 33)	*p*-Value
Target Lesion Failure, *n* (%)	4 (3.4)	4 (4.7)	0 (0)	0.205
All-Cause Death, *n* (%)	8 (7)	6 (7.3)	2 (6.3)	0.841
Cardiac Death, *n* (%)	2 (2)	2 (2.5)	0 (0)	0.367
Target Vessel MI, *n* (%)	0 (0)	0 (0)	0 (0)	
Non-Target-Vessel MI, *n* (%)	1 (1)	0 (0)	1 (3.4)	
Stroke, *n* (%)	4 (3.5)	3 (3.7)	1 (3.1)	0.881
TLR, *n* (%)	2 (2)	2 (2.8)	0 (0)	0.361
TVR, *n* (%)	2 (2)	2 (2.8)	0 (0)	0.361
AKI, *n* (%)	2 (2)	1 (1.2)	1 (3.1)	0.492
BARC Bleeding				0.740
BARC 1, *n* (%)	2 (2.7)	2 (3.6)	0 (0)	
BARC 2, *n* (%)	4 (5.3)	3 (5.5)	1 (5)	
BARC 3A, *n* (%)	1 (1.3)	1 (1.8)	0 (0)	
BARC 3C, *n* (%)	2 (2.7)	2 (3.6)	0 (0)	
BARC 5, *n* (%)	0 (0)	0 (0)	0 (0)	

Values are *n* (%), mean SD or median with interquartile range. Target lesion failure was defined as a composite of cardiovascular death, clinically driven TLR and target vessel MI. AKI: acute kidney injury; BARC: Bleeding Academic Research Consortium; MI: myocardial infarction; TLR: target lesion revascularization; TVR: target vessel revascularization.

**Table 4:** Outcomes during follow-up based on the microcirculation status evaluated as AMR.

Follow-Up Outcomes				
	Overall (*n* = 42)	AMR < 3.7 (*n* = 8)	AMR ≥ 3.7 (*n* = 34)	*p*-Value
Target Lesion Failure, *n* (%)	2 (4.7)	1 (12.5)	1 (2.9)	0.253
Death, *n* (%)	2 (4.8)	0 (0)	2 (5.9)	0.841
Cardiac Death, *n* (%)	0 (0)	0 (0)	0 (0)	
Target Vessel MI, *n* (%)	0 (0)	0 (0)	0 (0)	
Non-Target-Vessel MI, *n* (%)	0 (0)	0 (0)	0 (0)	
Stroke, *n* (%)	1 (2.4)	0 (0)	1 (3.1)	0.623
TLR, *n* (%)	2 (5.3)	1 (16.7)	1 (3.1)	0.173
TVR, *n* (%)	2 (5.3)	1 (16.7)	1 (3.1)	0.173

Values are *n* (%), mean SD or median with interquartile range. Target lesion failure was defined as a composite of cardiovascular death, clinically driven TLR and target vessel MI. AMR: angiographic microvascular resistance; MI: myocardial infarction; TLR: target lesion revascularization; TVR: target vessel revascularization.

## Data Availability

The original contributions presented in this study are included in the article. Further inquiries can be directed to the corresponding authors.

## Abbreviations

AMR	Angiographic Microvascular Resistance
BARC	Bleeding Academic Research Consortium
DCB	Drug-Coated Balloon
DES	Drug-Eluting Stent
IMR	Index of Microvascular Resistance
PCI	Percutaneous Coronary Intervention
pPCI	Primary Percutaneous Coronary Intervention
STEMI	ST-Elevation Myocardial Infarction
TLF	Target Lesion Failure
